# Expression network analysis of bovine skin infested with *Rhipicephalus australis* identifies pro-inflammatory genes contributing to tick susceptibility

**DOI:** 10.1038/s41598-024-54577-w

**Published:** 2024-02-23

**Authors:** Emily F. Mantilla Valdivieso, Elizabeth M. Ross, Ali Raza, Loan Nguyen, Ben J. Hayes, Nicholas N. Jonsson, Peter James, Ala E. Tabor

**Affiliations:** 1 Queensland Alliance for Agriculture and Food Innovation, Centre for Animal Science, The University of Queensland, St Lucia, QLD 4072 Australia; 2 Institute of Biodiversity One Health and Veterinary Medicine, University of Glasgow, Glasgow, G61 1QH UK; 3 School of Chemistry and Molecular Biosciences, The University of Queensland, St Lucia, QLD 4072 Australia

**Keywords:** Gene expression, Cattle, Bovine, Host resistance, RNA-Seq, Computational biology and bioinformatics, Immunology

## Abstract

The skin is the primary feeding site of ticks that infest livestock animals such as cattle. The highly specialised functions of skin at the molecular level may be a factor contributing to variation in susceptibility to tick infestation; but these remain to be well defined. The aim of this study was to investigate the bovine skin transcriptomic profiles of tick-naïve and tick-infested cattle and to uncover the gene expression networks that influence contrasting phenotypes of host resistance to ticks. RNA-Seq data was obtained from skin of Brangus cattle with high (n = 5) and low (n = 6) host resistance at 0 and 12 weeks following artificial tick challenge with *Rhipicephalus australis* larvae. No differentially expressed genes were detected pre-infestation between high and low resistance groups, but at 12-weeks there were 229 differentially expressed genes (DEGs; FDR < 0.05), of which 212 were the target of at least 1866 transcription factors (TFs) expressed in skin. Regulatory impact factor (RIF) analysis identified 158 significant TFs (*P* < 0.05) of which *GRHL3*, and *DTX1* were also DEGs in the experiment. Gene term enrichment showed the significant TFs and DEGs were enriched in processes related to immune response and biological pathways related to host response to infectious diseases. Interferon Type 1-stimulated genes, including *MX2*, *ISG15*, *MX1*, *OAS2* were upregulated in low host resistance steers after repeated tick challenge, suggesting dysregulated wound healing and chronic inflammatory skin processes contributing to host susceptibility to ticks. The present study provides an assessment of the bovine skin transcriptome before and after repeated tick challenge and shows that the up-regulation of pro-inflammatory genes is a prominent feature in the skin of tick-susceptible animals. In addition, the identification of transcription factors with high regulatory impact provides insights into the potentially meaningful gene–gene interactions involved in the variation of phenotypes of bovine host resistance to ticks.

## Introduction

The cattle tick *Rhipicephalus microplus* is a species complex distributed across tropical and subtropical regions worldwide, consisting of *Rhipicephalus australis*, *Rhipicephalus annulatus*, and three mitochondrial clades (A, B, C) of *R. microplus*^1,2^. Eighty percent of the world’s cattle population are at risk of cattle tick infestation, which causes estimated annual losses of US$ 22–30 billion^3^. Heavy tick infestation causes blood loss, anaemia, a decrease in live weight, and diminished reproductive performance. Cattle ticks are also responsible for the transmission of babesiosis and anaplasmosis, two highly infectious and potentially fatal diseases of cattle^4^.

*Rhipicephalus microplus* are primarily single-host parasites with an average life cycle duration of 21 days. Ticks start on the host as larvae, feed on blood, moult to nymphs, and then moult to adults. Male ticks tend to remain on the skin of the host, feeding occasionally, whereas female ticks feed until fully engorged and then proceed to disengage from the host, fall to the ground, lay eggs, and die^5,6^. Ticks have chemosensory structures that allow them to seek a suitable host and select attachment sites on the host’s skin. A sharp projection called hypostome serves as a food canal and passage of proteolytic enzymes into the host. Tick saliva can digest host tissues, dilate skin capillaries, prevent blood clotting, and stimulate the host’s immune system^7,8^.

Studies on local immune responses and gene expression of skin have revealed important aspects of bovine resistance to ectoparasites. Histology of tick-infested skin showed infiltration with eosinophils and neutrophils, mast cell disruption, histamine release, altered coagulation, and formation of intradermal vesicles^9,10^. The number and type of leukocyte populations (CD3^+^, γδ T cells, CD4^+^, CD8^+^, CD25^+^) at the larval attachment site appear to differ between Holstein and Brahman cattle^11,12^, but not significantly between Santa-Gertrudis cattle of high and low resistance^13^. A microarray study showed that the skin gene expression profiles of cattle were altered in the 24 h following tick infestation, primarily for keratin and mitochondrial genes^14^. Piper et al.^15^ contrasted the gene expression of Holstein and Brahman cattle and reported the upregulation of cytokine genes and inflammatory processes in the tick susceptible breed (Holsteins), whereas upregulation of extracellular matrix genes and tissue remodelling was found in the resistant breed (Brahman).

Using RNA-seq, Franzin et al.^16^ reported the expression of pro-inflammatory chemokines and cytokines that participate in the recruitment of granulocytes and T lymphocytes in tick-infested skin which is a delayed response in tick susceptible hosts. They also found higher expression of genes encoding volatile compounds in tick susceptible Holsteins (*Bos taurus taurus*) cattle when comparing tick resistant Nelore cattle *(Bos taurus indicus*). Another RNA-seq study that profiled the skin of infested Braford cattle (50% *Bos taurus taurus/Bos taurus indicus*) found leukocyte chemotaxis to be overrepresented in both resistant and susceptible cattle, whereas skin degradation and remodelling were upregulated in resistant hosts and proposed the Wnt-signalling pathway to be relevant for host resistance^17^. Making firm conclusions from gene-expression studies is challenging due to the variation introduced by the type of breed, previous exposure of the animals to ticks, and the methodology used for phenotypic classification of resistance, as reviewed in Tabor et al.^18^. In addition, knowledge of the expression patterns of skin from more composite breeds with variable phenotypes of tick resistance is still lacking.

The aims of this study were (1) to evaluate the skin transcriptome profiles of composite (Brangus) cattle using RNA-seq to identify significant differentially expressed genes and transcription factors involved in the regulation of responses against tick infestation in animals of high and low host resistance to ticks, and (2) to describe co-expression networks to predict important regulatory genes at the skin level which might help explain the complex biological processes underlying this economically relevant trait.

## Methods

### Animals and phenotypes of host resistance

This study was approved by the Animal Ethics Unit at The University of Queensland (certificate number QAAFI/469/18). Thirty tick-naïve Brangus steers (~ 9 months old) were artificially infested with ~ 10,000 *Rhipicephalus australis* larvae (pathogen-free ‘non-resistant field strain’, Queensland Department of Agriculture & Fisheries, Biosecurity Tick Colony) for 12 consecutive weeks and the individual resistance level was determined with mean tick scores as previously described^19^. For this study, the top 6 animals were assigned the High host resistance phenotype (HR), and the bottom 6 animals were assigned the Low resistance phenotype (LR).

### Collection of skin biopsies

Each animal was restrained in a crush and administered an epidural injection using 3 mL of lignocaine HCl 20 mg/mL (Troy Animal Healthcare, Australia) to desensitize the tail-head and escutcheon area. Skin biopsies were collected from the peri-anal area with 6 mm punches at 24 h before the first larval challenge (week 0/T0) and then at 24 h after the last tick challenge on week 12 (T12) of the experimental trial. Immediately after collection, the pre-infestation samples were frozen in liquid nitrogen, however, to improve RNA quality, the post-infestation samples were collected directly into RNA*later* (ThermoFisher, USA). All samples were kept at − 80 °C until resistance phenotype ranking was completed.

### Genomic estimate of *Bos indicus* content (BIC)

To test if the levels of host resistance were correlated with the amount of *B. indicus* content in the breed, genotyping data was generated. The tail hairs of all 30 Brangus steers were collected and submitted to Neogen Australasia in Gatton, Australia for DNA extraction and genotyping with the GeneSeek^®^ Genomic Profiler™ Bovine 50 K chip (GGP 50 K). The *Bos indicus* content (BIC) was calculated using a 35 K subset of the 50 K array by comparing each animal to a large purebred *B. indicus* dataset^20^. Using a GBLUP model, phenotypes were assigned as 1 for *B. indicus* and 0 if not and the effect of each SNP was back- solved^21^. Prediction equations for *B. indicus* purebred were then used to estimate BIC in the steers^22^. Of the 30 samples, 1 failed QC and could not be re-processed. As such, the BIC values of 29 steers ranging between 25 and 49% were further used to perform a Pearson correlation analysis with mean tick score data collected from the infestation trial.

### RNA isolation and sequencing

RNA was isolated from the frozen tissues of six steers with high host resistance (HR), and six steers with low host resistance (LR) before infestation (T0) and 12 weeks after infestation (T12), hereafter referred to as LR-T0, HR-T0, LR-T12, and HR-T12. Each skin biopsy was processed as fast as possible using a pre-chilled hammer and frozen metal block^23^. Then, the pulverized tissues were transferred to 1200 µL of QIAzol lysis reagent (QIAGEN, USA) in 2 ml tubes containing a bead mix (5 mm stainless steel + 3 mm glass beads) and homogenized in the TissueLyser II (QIAGEN, USA) at 30 Hz for 30 s twice. Skin RNA isolation was performed with the miRNeasy mini kit (QIAGEN, USA) as per manufacturer’s instructions. RNA samples were treated with DNA-free™ DNAse (Life Technologies, USA) and RNA was quantified in the Nanodrop 2000 Instrument (ThermoFisher, USA). The RNA integrity analysis was performed using a 2100 Bioanalyzer Instrument (Agilent Technologies, USA) by the Institute for Molecular Biosciences Sequencing Facility in St. Lucia, Australia. A total of 24 cDNA libraries were prepared with the TruSeq Stranded Total RNA kit with Ribo-depletion (Illumina, USA) and sequenced as 100 bp paired-end reads on one full S1 200 cycle flowcell on the NovaSeq 6000 sequencer (Illumina, USA). The Illumina bcl2fastq 2.20.0.422 pipeline was used to generate the sequence data. Library preparation and sequencing was performed by the Australian Genome Research Facility (AGRF) in Melbourne, Australia.

### Differential gene expression analysis

**Figure 1:**
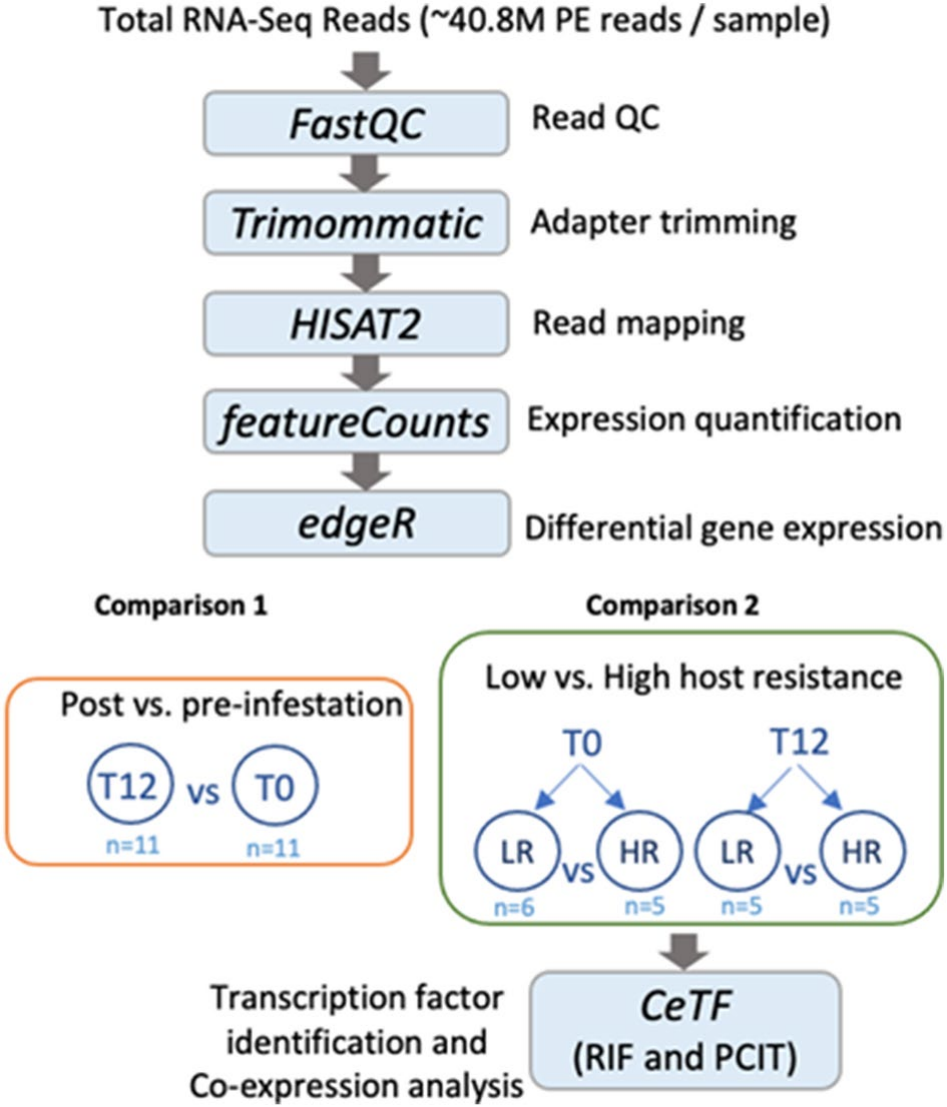
Schematic representation of the bioinformatic pipeline implemented in the analysis of total RNA-Seq data from skin of Brangus steers before and after tick challenge. Shaded boxes represent software packages. The timepoint tick scores, mean tick scores, RNA integrity numbers (RIN), and *Bos indicus* content (BIC) values for the selected high and low resistant animals are summarised in Supplementary File 1.

Total RNA-Seq data processing and differential gene expression were performed using the bioinformatic pipeline detailed previously^19^ and as represented in Fig. 1. Briefly, read quality control was performed with FastQC tool (version 0.11.4)^24^ and adapters were removed with Trimmomatic tool (version 0.35)^25^ using parameters for paired-end reads. Reads were aligned to the genome assembly *Bos taurus* ARS-UCD1.2 using HISAT2 (Galaxy version 2.1.0 + galaxy6)^26^. Gene count matrices were generated with featureCounts tool (Galaxy Version 1.6.4)^27^ and fed into RStudio (RStudio Team, 2020) for differential gene expression analysis with *edgeR* Bioconductor package^28,29^. Pairwise comparisons were made between post- and pre-infestation samples (T12-vs-T0), as well as between low and high host resistance samples within each timepoint (LR_T0_-vs-HR_T0_; LR_T12_-vs-HR_T12_).

To identify genes with differential expression after tick exposure, the data was fitted in *edgeR* using the quasi-likelihood F-test and the design matrix y ~ IW + MTS + BIC + RIN, where y is the corrected count for that gene/transcript and IW is infestation week timepoint fitted as a factor with two levels: 1 for post-infestation and 0 for pre-infestation. The covariates of mean tick score (MTS), *B. indicus* content (BIC), sample RNA Integrity Number (RIN) value were included to account for the effect of individual tick burden, indicine proportion, and level of sample degradation, respectively^19^. One HR animal that was not genotyped (no BIC available) was dropped from the model, and thus, the pairwise contrast was performed with 11 animals in each group. Significant differentially expressed genes (DEGs) were identified with a false discovery rate (FDR) threshold at FDR < 5%.

To identify the genes with differential expression due to host resistance phenotype, the datasets were fitted with the likelihood ratio test (edgeRLRT) using the design matrix:

$$ {\text{y }} \sim {\text{MTS}} + {\text{BIC}} + {\text{RIN}} $$

For the pre-infestation data, whereas the design matrix

$$ {\text{y }}\sim {\text{MTS}} + {\text{TPS}} + {\text{BIC}} + {\text{RIN}} $$

was used for the post-infestation data. In these models, y is the corrected count for that gene/transcript, the mean tick score (MTS) was fitted as a continuous variable representing the estimated tick burden while accounting for the effects of timepoint tick score (TPS, in post-infestation data only), *B. indicus* content (BIC) and sample RIN value (RIN)^19^. One HR animal was missing a BIC value, whereas one LR steer was missing a TPS score at timepoint T12 and for this reason they were dropped from the models. Therefore, the two pairwise contrasts were LR_T0_ (n = 6) vs. HR_T0_ (n = 5) and LR_T12_ (n = 5) vs. HR_T12_ (n = 5). Significant DEGs were identified with a threshold at FDR < 5%. The data for all covariates implemented in the models is provided in Supplementary File 1.

### Functional enrichment analysis

Over-representation analysis (ORA) of Gene Ontology (GO) terms for biological processes and KEGG (Kyoto Encyclopedia Genes and Genomes) pathways were performed with the *clusterProfiler* R package^30^ using filtered lists of significant DEGs (FDR < 0.05 and |logFC|> 1) and TFs (from RIF analysis) from T12-vs-T0 and LR-vs-HR at T12 comparisons. Additional filtering of highly redundant parent–child terms in the ORA-GO output was applied with the *simplify* function with a 0.7 threshold value. Graphics were created with dot plot and category network plot functions from this package. KEGG pathway graphs were created with the *Pathview* R package^31^ for *Bos taurus* organism.

### Co-expression analysis using RIF and PCIT analysis

Regulatory Impact Factor (RIF)^32^ and Partial Correlation and Information Theory (PCIT) analyses^33^ were implemented using the CeTF package in RStudio^34^ with the *RIF* and *PCIT* functions, respectively. The filtered and normalised gene expression matrices (log counts per million/logCPMs) obtained in the DE analyses performed with edgeR were used as input to the package. The list of TFs and cofactors were obtained from the AnimalTFDB bovine database^35^ to identify expressed TFs in the skin data. Briefly, RIF1 metric captures TFs showing differential connectivity to DEGs found between high and low resistance phenotypes, whereas RIF2 focuses on TFs showing evidence as predictors of change in abundance of these DEGs. The TFs with RIF1 and RIF2 (z-scores) above ± 1.96 standard deviation from the mean (corresponding to *p* < 0.05) were considered significant^32,33^. The PCIT analysis was used to determine the significance of the correlation between two nodes (regulatory interaction) after accounting for all other nodes in the network. Connections between nodes above the Pearsons correlation threshold |r|> 0.9 were considered strong evidence of interaction between transcripts^32,33^. PCIT analyses were run separately for the HR and LR conditions and compared to identify the most differentially connected genes in their networks which may be relevant to phenotype. Co-expression networks were visualised in Cytoscape (version 3.8.2)^36^.

The DEGs that were common to this study and those described previously in the RNA-Seq study of bovine peripheral blood leukocytes^19^ were identified to validate the potential target genes of TFs highlighted in the co-expression analysis. 

### Ethics approval

The study was conducted under approval of The University of Queensland Animal Ethics for project “Biomarkers and accurate phenotyping for selecting resistance to ectoparasites” with certificate number QAAFI/469/18. All methods were carried out in accordance with the relevant guidelines and regulations. All methods were reported in accordance with the ARRIVE guidelines (Animal Research: Reporting of In Vivo Experiments).

## Results

### Differentially expressed genes in skin at pre- and post-infestation

**Figure 2:**
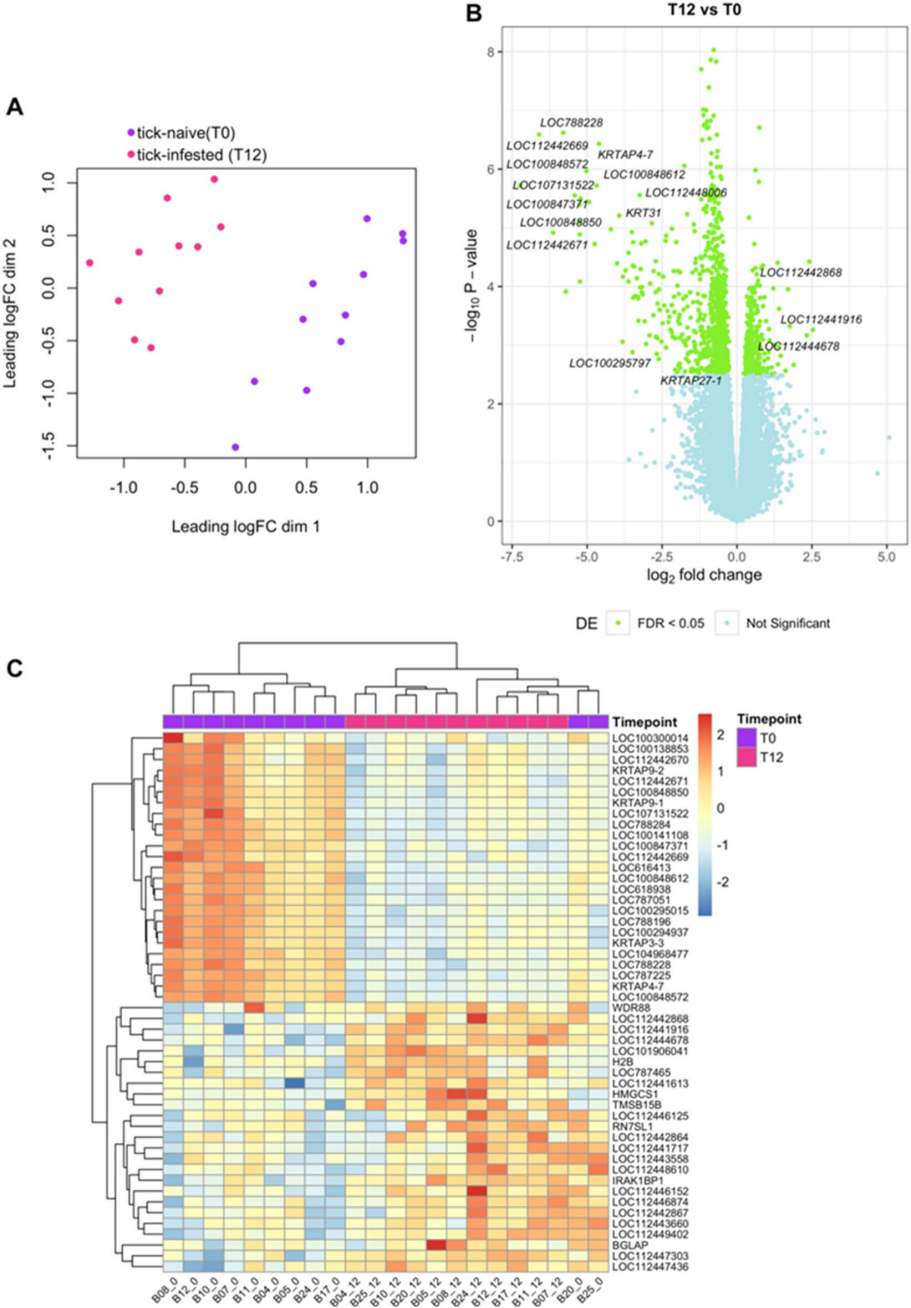
Differentially expressed genes (DEGs) in skin from tick-infested compared to tick-naïve steers. (**A**). Multidimensional scaling (MDS) plot shows clustering of samples according to sampling timepoint (T0: tick-naive; T12: week12 post-initial infestation). (**B**) Volcano plot of DEGs where x-axis represents fold change and y-axis represents statistical significance. Gene symbols are shown for DEGs with fold change |logFC|> 2 and FDR < 0.05. (**C**) Heatmap of the top 50 DEGs (25 most upregulated and 25 most downregulated) showing gene expression levels (logCPM) across samples from each timepoint.

To investigate the effect of tick infestation on Brangus skin of both high and low host resistance, a differential gene expression model was fitted in edgeR to compare samples from T12 (week-12 tick-infested, n = 11) to T0 (tick-naïve, n = 11) accounting for other covariates including host resistance (defined by MTS), *B. indicus* content (BIC) and RNA quality value (RIN). EdgeR identified a total of 17,387 genes expressed in the skin of Brangus steers. Multidimensional scaling analysis showed that T0 and T12 samples clustered separately, as expected (Fig. 2A). There were 1085 genes differentially expressed in the comparison of post- and pre-infestation skin samples (FDR < 0.05). Of these, 322 genes were upregulated and 763 genes were downregulated (Fig. 2B). The top upregulated skin DEGs (logFC > 2) were *LOC112441916* (U5 spliceosomal RNA), *LOC112442868* (small nucleolar RNA U3) and *LOC112444678* (small nucleolar RNA U13). Larger fold changes of differential expression were observed among the top downregulated genes (− 7 < logFC < − 2), most of which were annotated as keratin-associated proteins such as *KRTAP9-2* (keratin associated protein 9–2) and *KRTAP9-1* (keratin associated protein 9–1). A heatmap of the top 50 DEGs in post- vs. pre-infested Brangus skin samples is shown in Fig. 2C.

### Differentially expressed genes in skin of low vs. high host resistance steers

**Figure 3:**
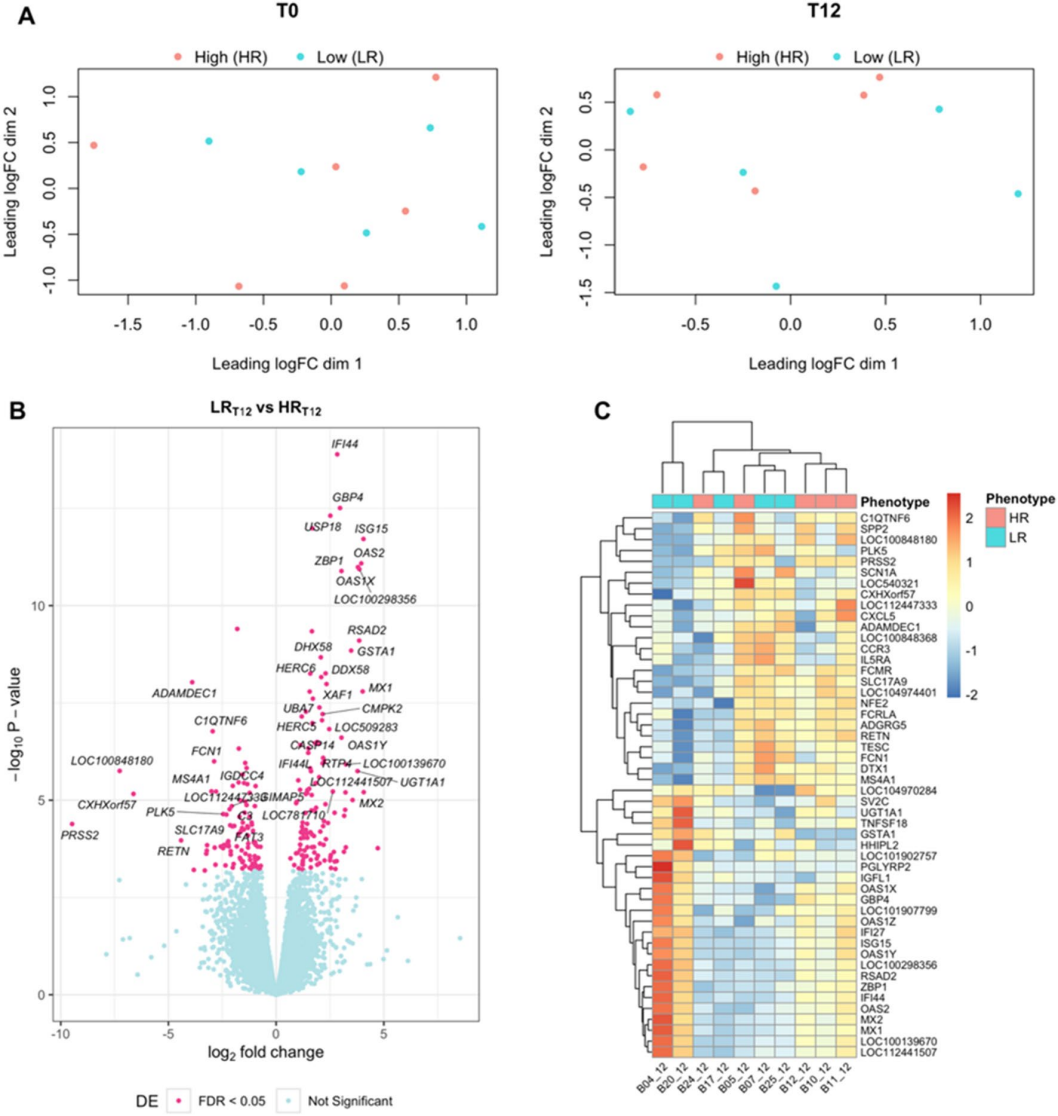
Differentially expressed genes in skin samples of low compared to high host resistance steers. (**A**) MDS plot shows clustering of samples according to phenotype within sampling timepoint (T0: tick-naive; T12: week12 post-initial infestation). (**B**) Volcano plot of DEGs in T12 comparison where x-axis represents fold change and y-axis represents statistical significance. Symbols are shown for DEGs with fold change |logFC|> 2 and FDR < 0.05. (**C**) Heatmap of the top 50 DEGs (25 most upregulated and 25 most downregulated) showing gene expression levels (logCPM) across samples from each timepoint.

To identify differentially expressed genes between host resistance phenotypes within each of the two observed timepoints, the edgeR gene expression models compared low (LR) and high (HR) host resistance steers as defined by the mean tick score (MTS), accounting for the effect of BIC and RIN covariates, also considering timepoint tick score (TPS) as a covariate only in tick-infested sample sets. The resulting comparison between pre-infestation datasets was defined as LR_T0_ (n = 5) vs. HR_T0_ (n = 6), whereas for post-infestation datasets was defined as LR_T12_ (n = 5) vs. HR_T12_ (n = 5). The total number of expressed genes was 17,566 and 17,512 genes for T0 and T12 comparisons, respectively. The Multidimensional scaling analysis showed that samples did not segregate well according to host resistance phenotype (Fig. 3A). Additionally at pre-infestation, there were no significant genes that were differentially expressed (FDR < 0.05) in the skin of low vs. high host resistance steers. However, in the post-infestation group comparison, there were 229 significant skin DEGs of which 128 were upregulated and 101 downregulated in low vs. high host resistance steers (Fig. 3B). The top 5 upregulated genes (logFC > 3.9) were *LOC104970284* (major allergen I polypeptide chain 1-like), *MX2* (MX dynamin like GTPase 2), *ISG15* (ISG15 ubiquitin like modifier), *MX1* (MX dynamin like GTPase 1), and *OAS2* (2'-5'-oligoadenylate synthetase 2). The top 5 downregulated genes (logFC < -3.9) were *PRSS2* (serine protease 2), *LOC100848180* (uncharacterized), *RETN* (resistin), *ADAMDEC1* (ADAM-like, decysin 1), *FCRLA* (Fc receptor like A). A heatmap of the top 50 skin DEGs in low vs. high host resistance phenotypes before and after tick challenge is shown in Fig. 3C.

Notably, the expression of three genes including *HSPG2* (heparan sulfate proteoglycan 2), *COL4A2* (collagen type IV alpha 2 chain) and *LTBP3* (latent transforming growth factor beta binding protein 3) was captured as significantly different in both intra- and inter-group comparisons (Table 1).

**Table 1:** Differentially expressed genes (FDR < 0.05) in skin of Brangus steers identified by both infestation timepoint and host resistance phenotype comparisons.

Symbol	Name	Timepoint (T12-vs-T0)^a^	Phenotype (LR_T12_-vs-HR_T12_)^b^
*HSPG2*	Heparan sulfate proteoglycan 2	− 1.17	− 1.00
*COL4A2*	Collagen type IV alpha 2 chain	− 1.19	− 1.13
*LTBP3*	Latent transforming growth factor beta binding protein 3	− 1.23	− 1.23

^a^T12-vs-T0 represents the fold change in gene expression between 12-week tick-exposed and tick-naïve steers.

^b^LR_T12_-vs-HR_T12_ represents the fold change in gene expression between low (LR) and high (HR) host resistance steers at 12 weeks post-initial infestation.

### Identification of regulators of differential expression in skin of tick-infested steers and their enriched biological processes

The potentially meaningful gene regulatory networks and transcription factors (TFs) in the Brangus skin transcriptome data were identified through the RIF algorithm and PCIT analysis. Briefly, RIF1 captures TFs showing differential connectivity to DEGs between high and low resistance phenotypes, whereas RIF2 focuses on TFs showing evidence as predictors of change in abundance of these DEGs. The PCIT algorithm determines the significance of the correlation between two nodes after accounting for all other nodes in a network. Strong correlations between genes (|PCIT|> 0.9) were evidence of interactions between transcripts.

**Figure 4:**
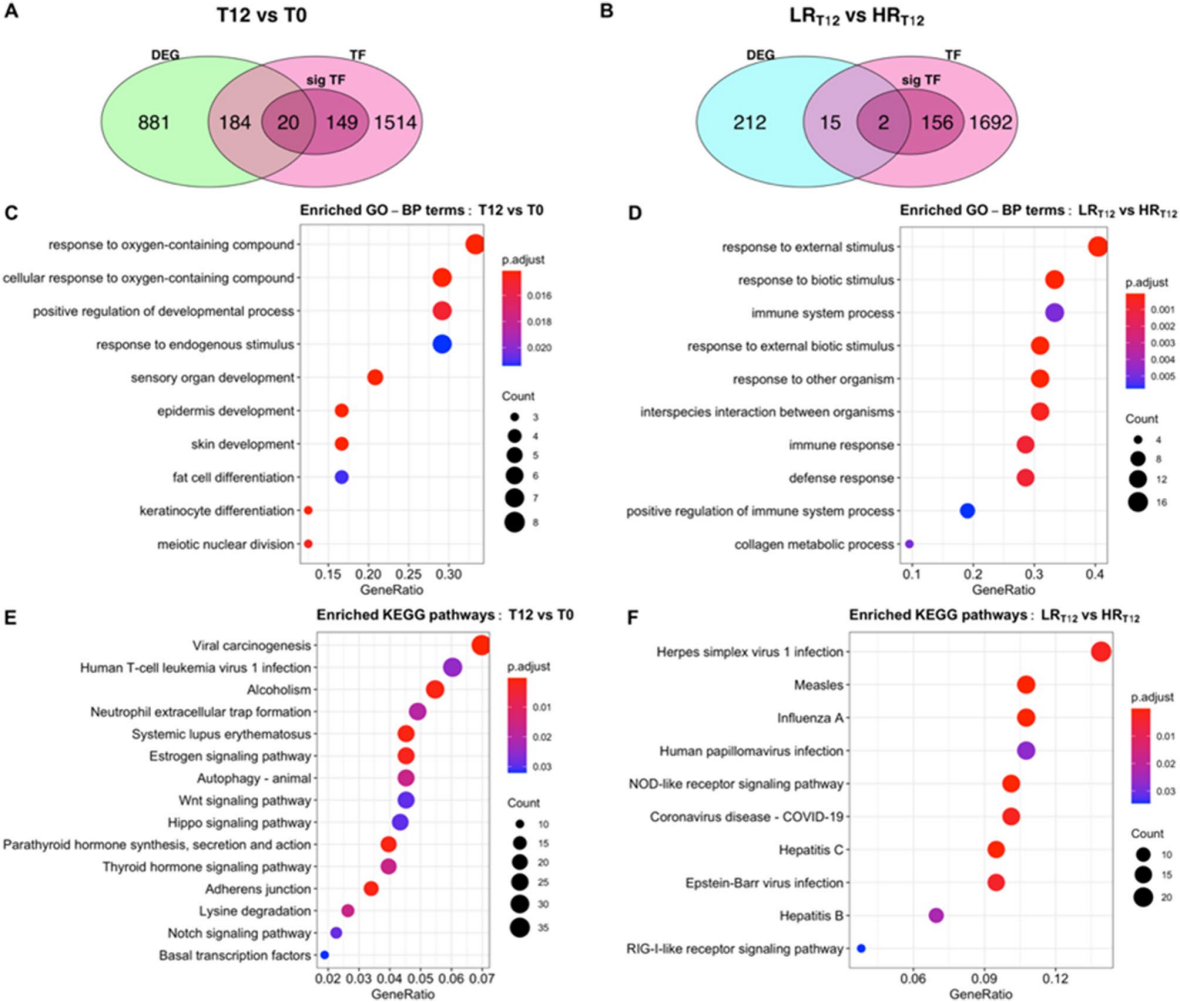
Distribution of significant DEGs and TFs in skin of Brangus steers and their functional enrichment. Venn diagram showing the distribution of genes identified as DEG, expressed TFs, and significant TFs (from RIF analysis) in the comparison of (**A**) tick-infested vs. tick-naïve steers (T12-vs-T0) and (**B**) high vs. low host resistance steers at T12 (LR_T12_-vs-HR_T12_). Enriched GO biological process terms in skin DEGs and TFs (FDR < 0.05, RIF metrics > 1.96, logFC > 1) from (**C**) T12-vs-T0 timepoint comparison and (**D**) LR_T12_-vs-HR_T12_ phenotype comparison. Enriched KEGG pathways in skin DEGs and TFs (FDR < 0.05, RIF metrics > 1.96) from (**E**) T12-vs-T0 timepoint comparison and (**F**) LR_T12_-vs-HR_T12_ phenotype comparison. The dot colour represents significance of the term (p-adjusted < 0.05), and dot size (GeneRatio) represents the number of significant genes in that pathway.

Using the Animal Transcription Factor Database (AnimalTFDB 3.0) it was found that 1866 genes expressed in the skin of Brangus steers were TFs. The RIF analysis showed that 169 of these TFs could be significant predictors (P-value < 0.05) for the change in abundance of 881 DEGs detected in post- vs. pre-infested skin samples (Fig. 4A). Additionally, it was found that 158 TFs could also be significant predictors for the change in abundance of 212 skin DEGs detected in the low vs. high host resistance steers (Fig. 4B). There were 22 genes identified as significant by both RIF and DE analysis (Fig. 4A,B, Table 2) which may be of interest for further characterization of regulators of the skin response to tick infestation.

**Table 2:** Top-ranking transcription factors identified as significant by regulatory impact factor metrics (RIF1 and RIF2, P < 0.05) and differential gene expression (FDR < 0.05) in timepoint and phenotype comparisons.

Comparison	TF symbol	Name	logFC^c^	FDR^d^	RIF1^a^	RIF2^b^
Timepoint (T12-vs-T0)	RNF168	Ring finger protein 168	0.38	1.12E − 02	2.66	0.01
	SP3	Sp3 transcription factor	0.31	4.77E − 02	1.79	2.17
	ZFP62	ZFP62 zinc finger protein	0.30	4.48E − 02	3.06	0.32
	MED13L	Mediator complex subunit 13L	− 0.29	4.16E − 02	2.43	− 0.82
	PBX1	PBX homeobox 1	− 0.35	4.40E − 02	2.20	− 0.54
	ZNF609	Zinc finger protein 609	− 0.35	2.13E − 02	0.54	2.01
	BTG1	BTG anti-proliferation factor 1	− 0.50	2.90E − 02	0.44	1.96
	MED25	Mediator complex subunit 25	− 0.50	2.83E − 02	0.09	2.09
	TRRAP	Transformation/transcription domain associated protein	− 0.54	1.56E − 03	0.02	2.47
	WIZ	WIZ zinc finger	− 0.55	2.37E − 02	− 0.09	2.55
	GLMP	Glycosylated lysosomal membrane protein	− 0.58	9.12E − 03	6.13	1.59
	BRD3	Bromodomain containing 3	− 0.58	3.06E − 02	0.67	2.22
	KDM4B	Lysine demethylase 4B	− 0.59	6.36E − 03	− 0.14	2.30
	ZNF710	Zinc finger protein 710	− 0.65	2.41E − 02	2.05	− 0.80
	SETD1A	SET domain containing 1A, histone lysine methyltransferase	− 0.76	1.97E − 03	1.04	2.60
	CIC	Capicua transcriptional repressor	− 0.85	2.75E − 03	− 0.80	2.00
	NCOR2	Nuclear receptor corepressor 2	− 0.92	2.63E − 04	− 0.15	2.14
	KMT2D	Lysine methyltransferase 2D	− 0.94	1.41E − 04	− 0.27	2.02
	CSRNP1	Cysteine and serine rich nuclear protein 1	− 1.03	3.45E − 03	0.55	− 2.12
	TFCP2L1	Transcription factor CP2 like 1	− 1.43	2.07E − 02	2.87	0.70
Phenotype (LR_12_-vs-HR_12_)	GRHL3	Grainyhead like transcription factor 3	1.24	4.80E − 02	0.50	− 2.68
	DTX1	Deltex E3 ubiquitin ligase 1	− 2.26	1.70E − 02	− 0.48	− 1.96

^a^RIF1 metric captures TF showing differential connectivity to DEGs found in the specified comparison. Significance value based on z-scores above 1.96 standard deviation from the mean.

^b^RIF2 metric captures TF showing evidence as predictors of change in abundance of the DEGs found in the specified comparison. Significance value based on z-scores above 1.96 standard deviation from the mean.

^c^FC = log2 fold change between compared conditions: Timepoint (T12-vs-T0) = tick-infested vs. tick-naive, and Phenotype (LR_T12_-vs-HR_T12_) = low vs. high host resistance at T12.

^d^FDR = False discovery rate corrected (Benjamini-Hochberg) p-values of gene expression.

The list of significant DEGs and TFs derived from the T12 vs T0 comparison was significantly enriched for GO biological process terms such as: response to oxygen-containing compound, response to endogenous stimulus, epidermis development, and keratinocyte differentiation (Fig. 4C). These genes were overrepresented in regulatory pathways including viral carcinogenesis and basal transcription factors, and also pathways related to cell proliferation and signalling processes including WnT, Hippo, and Notch signalling (Fig. 4E). Some top-ranking TFs such as *SETD1A* and *KMT2D* featured in the lysine degradation pathway, *NCOR2* in the Notch signalling pathway, and *MED13L* in thyroid hormone signalling pathway.

In contrast, for genes from the low resistant vs high resistant phenotype comparison at T12, most of the enriched biological processes were related to: response to external stimulus, immune system process, and, response to other organism (Fig. 4D). The corresponding enriched biological pathways were disease-related including “Measles” and “Herpes simplex virus 1 infection”, as well as immune signalling pathways such as NOD-like receptor and RIG-I-like receptor (Fig. 4F). Genes that were previously identified as highly upregulated in LR steers (*MX2*, *ISG15*, *MX1*, *OAS2*) and the highly downregulated *PRSS2* featured in several of the disease-related pathways. Similarly, *IRF7* (interferon regulatory factor 7) was the most notable TF gene to be enriched in several of these pathways and it was found to be also upregulated in the LR condition. Category network plots of the enriched GO and KEGG terms featuring the corresponding positively or negatively regulated genes are presented in Supplementary File 2.

### Co-expression of skin genes in relation to host resistance divergence

Co-expression networks were derived from 385 nodes consisting of the top-ranking TF genes and their DEG targets from the LR_T12_-vs-HR_T12_ comparison to predict the regulatory associations of the skin transcripts involved in host resistance to ticks in Brangus steers. Comparison of the individual networks showed that overall, the LR condition had a higher number of connections (85,550) than the HR network (3575). Additionally, transcription factors (DE or not) accounted for the highest percentage of connections in the co-expression networks, as expected given their regulatory roles, with 71% (6069/8550) and 72.5% (2594/3575) in LR and HR networks, respectively.

The five most connected potential regulators (from highest to lowest) in the LR network were *GRHL3, PCBD1*, *TEAD3*, *NR3C1*, and *SP3*, whereas in the HR network were *ZNF18*, *ARID4B*, *XPA*, *PRDM2*, and *TAF1C*. Among the most connected differentially expressed genes in the LR condition, there were *LOC100848368*, *CLEC11A*, *SERPINH1*, *CDSN*, as well as *GRHL3*. The most connected DEGs in the HR phenotype were *PARP9*, *LOC509283*, *DHX58*, *IFI44L, P4HA2*. The *GRHL3* gene was also found to be one of the most connected TF in both the LR and HR networks with 82 and 9 connections, respectively. The top 5 genes (DEG and/or TF) in the co-expression network of high and low host resistance phenotypes are listed in Table 3 with the number of connections detected by PCIT analysis, as well as the fold change (positive or negative) relative to the contrasting group.

**Table 3:** List of most co-expressed transcripts in HR and LR networks.

Network	Symbol	Name	No. of connections^a^	Fold change^b^	Category^c^
LR	LOC100848368	Uncharacterized LOC100848368	90	−	DEG
	CLEC11A	C-type lectin domain containing 11A	85	−	DEG
	GRHL3	Grainy head-like transcription factor 3	82	+	DEG TF
	PCBD1	Pterin-4 alpha-carbinolamine dehydratase 1	82	−	TF
	SERPINH1	Serpin family H member 1	82	−	DEG
	TEAD3	TEA domain transcription factor 3	82	+	TF
	CDSN	Corneodesmosin	81	+	DEG
	NR3C1	Nuclear receptor subfamily 3 group C member 1	78	−	TF
	SP3	Sp3 transcription factor	77	−	TF
HR	PARP9	Poly(ADP-ribose) polymerase family member 9	46	−	DEG
	LOC509283	E3 ubiquitin-protein ligase RNF213	42	−	DEG
	DHX58	DExH-box helicase 58	41	−	DEG
	ZNF18	Zinc finger protein 18	41	−	TF
	IFI44L	Interferon induced protein 44 like	40	−	DEG
	P4HA2	Prolyl 4-hydroxylase subunit alpha 2	40	+	DEG
	ARID4B	AT-rich interaction domain 4B	39	−	TF
	XPA	XPA, DNA damage recognition and repair factor	36	+	TF
	PRDM2	PR/SET domain 2	35	−	TF
	TAF1C	TATA-box binding protein associated factor, RNA polymerase I subunit C	35	+	TF

^a^Number of gene connections in a gene co-expression network determined by PCIT analysis. The significance of the connections is based on z-scores above 1.96 standard deviation from the mean.

^b^Fold Change indicates if the gene was downregulated gene ( −) or upregulated ( +) in the comparison of low vs. high host resistance groups at post-infestation.

^c^Category indicates if the gene was identified as significant by differential expression analysis (DEG; FDR < 0.05) and/or RIF analysis (TF; RIF metrics > 1.96).

### Comparison of skin and leukocyte DEGs

To further explore the relevance of DEGs as potential biomarkers of host resistance, the present study was compared to a leukocyte transcriptome dataset obtained from the same Brangus herd previously^19^. The DEG sets from similar experimental timepoints were compared to find relevant overlapping genes expressed in low vs. high host resistance steers at week 12 of post-infestation. This showed that 86 genes were expressed in both skin and leukocyte datasets. Of these, 44 were TFs and 42 were differentially expressed genes (FDR < 0.05). There were 3 genes that were differentially expressed between low and high host resistance comparisons in both studies, including *FCMR*, GPR82, and *LOC104974401*. Additionally, 14 DEGs were significantly correlated with more DEGs in the LR compared to the HR co-expression network including: *C3*, *CCR3*, *CYP27A1*. *FCN1*, *GIMAP5*, *IL5RA*, *KDELR3*, *RETN*, *RHOD*, *RIPOR2*, *SLC46A2*, *SLFN11*, *MEM263*, *TUBA4A*. These genes were also found to be differentially expressed in the leukocytes of Brangus steers exposed to 3 and 12 week of tick infestation compared to their naïve selves. Lastly, *TOB2* was one of the only TFs that was differentially expressed in both skin and leukocytes from Brangus steers across all post- vs. pre-infestation comparisons but was not DE among host resistance phenotypes. The list of relevant genes from the co-expression analysis that were also identified as DE in the Brangus leukocyte transcriptomic study is shown in Supplementary File 3.

## Discussion

Resistance against the cattle tick is primarily manifested at the skin level where tick attachment and feeding are restricted by a complex interplay of immune and non-immune mechanisms elicited by the bovine host^16,17,37^. Although it is widely recognized that resistance to ticks is higher in *B. indicus* breeds compared to *B. taurus* breeds, it is still a challenge to predict how well crossbred cattle (*B. indicus* x *B. taurus*) will perform in production systems that are at risk of tick infestations due to higher individual animal variation of host resistance. In addition to this, measuring host resistance to ticks in cattle herds is generally difficult and expensive. However, with the progress in high-throughput sequencing technologies that allow the profiling of the whole transcriptome of an organism it has become more feasible to dissect the underlying biology of this complex trait. The identification of predictive biomarkers of host resistance holds potential to sped up the identification of divergent phenotypes for this trait so that these can be used in selective breeding programs that include enhanced host resistance as part of the breeding goal. These approaches are expected to contribute to advances in tick control and reduce economic losses due to tick outbreaks^18,38^.

In our previous study, it was observed that tick-naïve Brangus steers displayed divergent phenotypes of host resistance following a 12-week tick infestation experiment with *R. australis* larvae, however it remained a challenge to unravel the transcriptional regulation of this complex trait from leukocyte gene expression data alone^19^. As such, to gain a more comprehensive understanding of the transcriptional profiles of Brangus steers of variable host resistance phenotype before and after tick infestation, the present study focused on the skin transcriptome. Skin samples from 5 high host resistance steers and 6 low host steers collected at two timepoints (T0 and T12) were profiled with RNA-Seq, robust differential expression analyses, functional enrichment analysis and co-expression analyses. Similarities in the experimental conditions and gene expression models between skin and leukocyte transcriptome data were essential to draw reliable conclusions from the predicted DEGs in the different tissue types of Brangus steers.

### Keratin genes downregulated in skin responses to tick infestation

The comparison of 12-week tick-infested skin samples (both HR and LR) to their pre-infestation matched samples showed many differentially expressed genes. One notable pattern was the downregulation of several keratin and keratin-associated protein genes (KRTAPs) in response to long-term tick infestation. The KRTAP multigene family is known to have an important role in supporting the mechanical strength and shape of hair by cross-linking the keratin intermediate filaments to build a hair shaft. Molecular diversity of KRTAPs and modulation of their expression appears to be relevant for hair size and shedding during coat changes in response to environmental adaptation^39^. In the present study at least ten genes including *KRTAP9-1* and *KRTAP9-2,* and *KRTAP9-1 like, KRTAP9-3 like, KRTAP9-7 like* and *KRTAP9-9-like*, and *LOC* genes, were among the most highly downregulated genes (|logFC > 5) in tick-infested (both HR and LR) steers. Similarly, a skin microarray study found uniform downregulation of keratin genes, including *KRTAP9-2*, in *B. taurus* cattle of high and low resistance at 24 h post-infestation with cattle ticks^14^. Although keratin gene expression is relevant for epidermal barrier maintenance, it is hypothesized that the downregulation of many keratin-associated genes may contribute to keratinocyte deformity, thus allowing the influx of immune cell populations to the tick bite site. From previous histological studies of tick-infested skin from Santa-Gertrudis cattle, it has been shown that a high number of neutrophils, eosinophils, γδ T cells, CD3 + , CD4 + , CD8 + , CD25 + T-cells are recruited to the larval attachment site compared to the non-infested controls^13^. As such it is possible that the low expression pattern of a keratin-associated gene in the skin of tick-infested HR and LR Brangus may also be in part due to the reduction in the cell types that express these genes due to the local immune response to tick infestation.

### Downregulation of skin homeostatic processes may enhance host inflammation and susceptibility to ticks

Skin differentiation and wound healing processes are tightly controlled processes and the deregulation or chronic activation of these can also lead to further tissue damage. For instance, Constantinoiu et al.^11^ reported that repeated infestation with ticks caused animals to develop skin lacerations at the site of tick application. This was also observed for the Brangus steers in this study between weeks 5 and 7 of the trial before animals were fully phenotyped. Researchers have previously observed that susceptible hosts concentrate more leukocytes at the tick attachment sites than resistant hosts, and this could represent a sign of pathology rather than a protective mechanism, thus causing further tissue damage that enhances tick feeding^11^.

Approximately 1.8% (20/1085) of the differentially expressed genes between tick-infested and tick-naïve steers were also identified by RIF analysis, suggesting that these transcripts have a potential regulatory role over the remaining 98.2% of DEGs associated with skin responses to tick infestation. The biological processes “cellular response to oxygen-containing compound” and “skin/epidermis development” were enriched by several downregulated genes that included three genes in common *MSX2*, *ZFP36, CYP26B. MSX2* (MX dynamin like GTPase 2) is part of the family of type I interferon (IFN)-stimulated genes and encodes an isoform of the MX protein which participates in the immune response. *ZFP36* encodes an RNA-binding protein member of the ZFP36 ring finger family which bind AU-rich elements within 3' untranslated regions (UTRs) to negatively regulate the post-transcriptional expression of targeted mRNAs^40^. Previous studies have found that *ZFP36* has an important role in the control of local skin inflammation by targeting cytokine genes, and loss of *ZFP36* in epidermal cells contributes to inflammatory phenotype in skin conditions such as psoriasis^41,42^. *CYP26B* encodes an enzyme of the cytochrome P450 family 26 involved in the degradation of retinoic acid, and the absence of CYP26B has been implicated in abnormal embryonic skin development and barrier dysfunction^43^. Furthermore, a study has shown that downregulation of *CYP26B* in skin fibroblasts may lead to imbalance in retinoic acid metabolism which induces mast cell- and P2X7-dependent dermatitis and exacerbates skin inflammation^44^. Taken together, these results suggest potential molecular disruptions to the skin-barrier homeostatic network that are caused by tick infestation and further highlights how loss of control over the regulation of skin inflammatory process could promote host susceptibility to ticks. This hypothesis is further supported by a recent Brangus skin proteomic study^45^ where it was observed that the skin of tick-resistant animals presents a higher abundance of proteins involved in the haemostasis triad including roles in blood coagulation, platelet aggregation and vasoconstriction.

### High expression of Type-1 IFN-inducible genes may contribute to inflammatory skin pathology in low host resistance steers.

The observed phenotypic divergence between high and low host resistance phenotypes among Brangus steers was further supported by the differences in their skin transcriptome profiles at 12-weeks after initial infestation. Biological processes related to host response to external biotic stimulus, immune response and viral disease-related pathways were significantly enriched among the DEGs and TFs from this comparison. Enrichment analysis suggested that type I interferon (IFN) responses are highly upregulated in cattle with low host resistance to ticks. This was determined by changes in expression for a group of eleven IFN-stimulated genes including *ISG15*, *OAS2*, *RSAD2*, *MX1*, *MX2, OAS1X, OAS1Y, OAS1Y, DDX58, DHX58, IFI44* and the transcription factors that regulate their expression such as *IRF7* (detected by RIF) and *STAT1* (detected by DE). IFN-stimulated genes are activated by interferon responses via the JAK/STAT signalling cascade leading to the production of antiviral effectors during immune response^46^. The function of these ISGs have been primarily studied in the context of innate immune response during viral infections (reviewed in Ref.^46^). For cattle in particular, high expression of ISGs (mainly *ISG15, MX1*, *MX2, OAS2*, *RSAD2*) has been reported in the peripheral blood immune cells of animals with bovine respiratory disease^47^, PPRV (*Peste des Petits Ruminants Virus*)^48^, and during early pregnancy in *B. indicus* heifers^49^. In a recent proteomic study^45^ it has also been shown that there is a higher abundance of several immunoglobulin-like proteins including C3 isoforms in the skin of tick-susceptible compared to resistant Brangus steers.

Although an activated interferon response may confer protection to viral infection, it is not yet clear how these responses may contribute to the rejection of ticks. From models for human autoimmune diseases such as systemic lupus erythematosus, it has been identified that type I IFN responses play a pathogenic role by increasing leukocyte recruitment to the skin via inflammatory cytokines, chemokines, and adhesion molecules, thereby inducing a cycle of the chronic skin inflammation^50^. Specifically, *IFIH1*, *STAT1*, and *IRF7* have been previously associated with lupus susceptibility loci and appear to be key regulators of the pathology of the disease^51^ and these three genes were also highly transcribed in steers with susceptibility to cattle ticks. The reduced expression of IFN-induced genes in high host resistance steers could also be an indication that resistant animals may have resolved inflammation at an earlier timepoint, while the susceptible animals are still under a chronic skin inflammatory state by week 12 post-initial infestation. It is therefore hypothesized that the ongoing activation of type I IFN responses in the LR phenotype may contribute to a pro-inflammatory state at the skin level which could be a major reason for the decreased resistance to tick infestation.

A notable observation in HR animals was the high upregulation of *RETN* (resistin) compared to LR animals at 12 weeks post-infestation. This gene also featured in the skin gene co-expression networks where it was highly correlated with changes in the LR animals compared to HR animals. Additionally, *RETN* was found to be downregulated in the leukocytes of Brangus steers exposed to 3- and 12-weeks post-infestation relative to pre-infestation^19^. The observation of this DEG in both leukocyte and skin transcriptome experiments suggest a potential for further investigation in bovine host resistance to ticks. *RETN* encodes a cysteine-rich protein called resistin which has been recently proposed as a novel host defence peptide of innate immunity^52^. Studies in human and mice models have reported the expression of resistin and resistin-like proteins in keratinocytes and sebaceous glands where these appear to provide protection against microbial infection in a vitamin-A-dependent manner^53,54^. However, as an adipocyte- and monocyte-derived cytokine, RETN also has been implicated in the induction of pro-inflammatory cytokines^52^. Besides *CXCL5*, there were no additional cytokine or chemokine genes with high expression in the HR phenotype that would be indicative of an inflammatory process in the skin of tick-resistant animals. In fact, the most upregulated gene in HR animals exposed to 12 weeks of tick infestation was *PRSS2* which encodes a serine proteinase with tissue-degrading function that could be relevant in tissue remodelling and leukocyte cell migration^55^. These results suggest that reduced local inflammation and ability to remodel skin tissue may contribute to higher levels of resistance to tick infestation as confirmed by a related proteomics study^45^. A potential protective role for *RETN* in the skin of resistant hosts is suggested, however, exact mechanisms of regulation for this gene and its activity against ectoparasites requires further investigation.

### Co-expression networks highlight a key regulator in epidermal differentiation

In this analysis, *GRHL3* (grainy head like transcription factor 3) emerged as a key regulator in cattle skin according to differential gene expression and RIF analysis, with a high RIF2 score indicating this gene is a potentially useful predictor of change in transcript abundance among the divergent host resistance phenotypes. *GRHL3* is a member of the CP2-like transcription factor family with functions in both epidermal differentiation during skin homeostasis and keratinocyte migration during wound healing^56^. Its capacity to drive distinct gene expression profiles that are unique for each keratinocyte functional state within the same cell-type allows for multiple transcriptional outputs, which is biologically relevant for a single regulator^57^. In this study, *GRHL3* showed altered expression as well as differential connectivity in co-expression networks for HR and LR conditions, which suggests that the keratinocytes from the divergent phenotypes may be committed to different stages of the wound healing process. Downregulation and low co-expression of this gene in the HR condition is consistent with the reduced need for tissue repair since highly resistant hosts were experiencing fewer tick attachments by week 12 of the experimental trial.

## Conclusions

Tick-infestation is associated with relatively lower expression of keratin-associated genes in both high and low resistant phenotypes. At 12 weeks post-infestation, the highly resistant animals show higher expression of genes with antimicrobial and tissue remodelling functions, whereas the steers with low host resistance show upregulation of INF-stimulated genes that participate in antiviral responses and contribute to skin inflammation. The gene expression profiles suggest the divergent phenotypes may be at different stages of the wound repair process given their significant differences in tick burden. Thus, the resistant animals may have resolved their inflammation at an earlier timepoint, while the susceptible animals are under a state of chronic skin inflammatory response. This observation has also been confirmed using proteomics analyses of skin from the same cohort where resistant cattle showed high abundances of proteins related to immunity, wound healing, and skin integrity. Higher co-expression of genes in steers with low resistance indicated that there may be more complex molecular mechanisms associated to the low resistance to ticks compared to the animals with high resistance phenotype. This skin transcriptome data is the first to integrate and compare gene expression results with the peripheral blood leukocytes from similar experimental conditions in Brangus steers.

### Supplementary Information

Supplementary Information.

## Data Availability

All relevant data are included in the manuscript and its Supplementary files. All RNA sequence data has been deposited in NCBI BioProject PRJNA802321.

## Abbreviations

BIC: *Bos indicus* content
DE: Differentially expressed
DEGs: Differentially expressed genes
FDR: False discovery rate
GO: Gene ontology
HR: High resistance
KEGG: Kyoto encyclopedia genes and genomes
LR: Low resistance
MTS: Mean tick score
MDS: Multidimensional scaling
ORA: Over-representation analysis
PCIT: Partial correlation and information theory
QC: Quality control
RIF: Regulatory impact factor
RIN: RNA integrity number
T(x): Timepoint (week) e.g. T12 + timepoint week 12; T0 = timepoint 0 (naïve)
TPS: Timepoint tick score
TFs: Transcription factors
