# Supplementary material for: Expression network analysis of bovine skin infested with Rhipicephalus australis identifies pro-inflammatory genes contributing to tick susceptibility
Source: Sci Rep. 2024 Feb 23;14:4419. doi: 10.1038/s41598-024-54577-w (PMC10884027; doi:10.1038/s41598-024-54577-w)
Supplement: Supplementary file 1 — Supplementary Information. [file 41598_2024_54577_MOESM1_ESM.pdf]

Supplementary File 1. Experimental design data used for modelling of gene expression of peripheral blood leukocytes. Table with sample IDs and their corresponding values for group (abbreviated), host resistance phenotype, infestation week timepoint, timepoint tick score, mean tick score, RIN value, and *Bos indicus* content.

**Sample ID**	**Group**	**Phenotype**	**Infestation week**	**TPS**	**MTS**	**RIN**	**BIC**
**B5_0**	HR	High	0	-	1	6.2	**0.43**
**B10_0**	HR	High	0	-	1.4	6.4	**0.39**
**B11_0**	HR	High	0	-	1.3	5.1	**0.36**
**B12_0**	HR	High	0	-	1	6.6	**0.49**
**B23_0**	HR	High	0	-	1.5	6.4	**NA**
**B24_0**	HR	High	0	-	1.5	6.6	**0.45**
**B4_0**	LR	Low	0	-	3.6	5.7	**0.35**
**B7_0**	LR	Low	0	-	4	5.7	**0.44**
**B8_0**	LR	Low	0	-	3.7	6.5	**0.33**
**B17_0**	LR	Low	0	-	4.1	6.1	**0.47**
**B20_0**	LR	Low	0	-	4.1	4.8	**0.41**
**B25_0**	LR	Low	0	-	4.5	5.3	**0.36**
**B5_12**	HR	High	12	1	1	7	**0.43**
**B10_0**	HR	High	12	1	1.4	7.4	**0.39**
**B11_12**	HR	High	12	1	1.3	7.7	**0.36**
**B12_12**	HR	High	12	1	1	7.3	**0.49**
**B23_12**	HR	High	12	1	1.5	6.4	**NA**
**B24_12**	HR	High	12	1	1.5	6.6	**0.45**
**B4_12**	LR	Low	12	3	3.6	7.5	**0.35**
**B7_12**	LR	Low	12	5	4	7.2	**0.44**
**B8_12**	LR	Low	12	NA	3.7	7.3	**0.33**
**B17_12**	LR	Low	12	5	4.1	7.3	**0.47**
**B20_12**	LR	Low	12	4	4.1	7.3	**0.41**
**B25_12**	LR	Low	12	5	4.5	7.2	**0.36**

LEGEND: Group & Phenotype: High host resistance (HR)/Low host resistance (LR); **Infestation week**: 0 = pre-infestation/week 0/T0 ; 3 = week 3 post-initial infestation / T3; 12 = week 12 post-initial infestation/ T0; **TPS**: Timepoint tick score measured by tick scoring method; **MTS**: Mean Tick Score calculated from scores collected between timepoints from week 8 to week 15 post-initial infestation; **RIN**: RNA Integrity Number determined from analysis with the 2100 Bioanalyser Instrument (Agilent Technologies, USA); **BIC**: *Bos indicus* content. Values represent percentage (%) written as decimal; **NA** = Not available

Supplementary File 2. Category gene network plot of top enriched GO Biological Process terms and KEGG pathways with their associated up- and down-regulated DEGs. A) Plot for enriched GO terms in the comparison of 12-week tick-infested vs. tick-naïve steers. B) Plot for enriched GO terms in the comparison of Low vs. High Host Resistance steers at 12-week post-initial infestation. C) Plot for enriched pathways in the comparison of 12-week tick-infested vs. tick-naïve steers. D) Plot for enriched pathways in the comparison of Low vs. High Host Resistance steers at 12-week post-initial infestation. Gene dot represents expression fold change (red=upregulated; blue= downregulated) and category dot size represents the number of genes annotated.

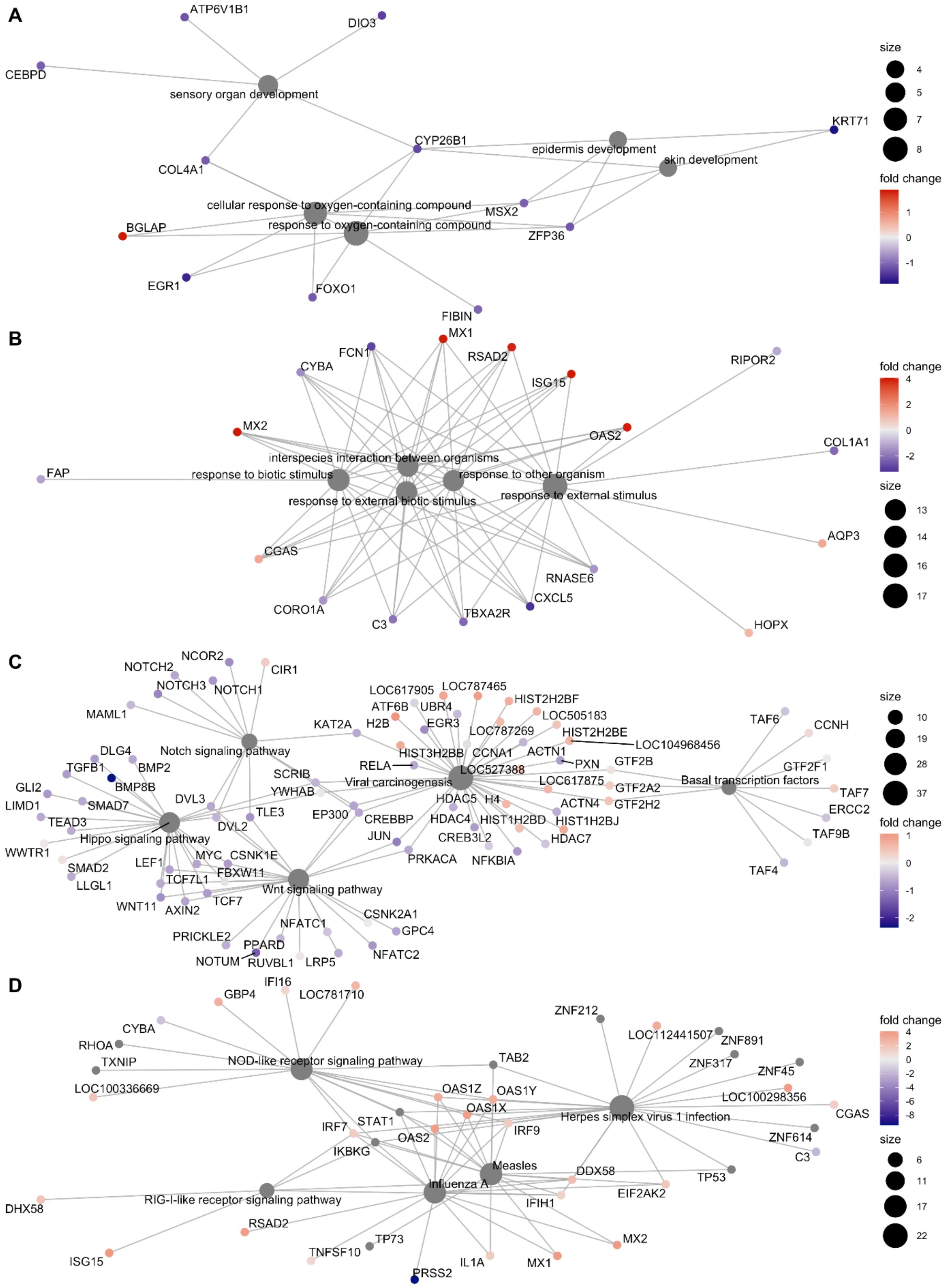
A
ATP6V1B1
DIO3
CEBPD
sensory organ development
KRT71
CYP26B1
epidermis development
skin development
COL4A1
cellular response to oxygen-containing compound
MSX2
BGLAP
response to oxygen-containing compound
ZFP36
EGR1
FOXO1
FIBIN
size
4
5
7
8
fold change
1
0
-1
B
MX1
FCN1
RSAD2
CYBA
RIPOR2
ISG15
MX2
OAS2
COL1A1
interspecies interaction between organisms
FAP
response to biotic stimulus
response to other organism
response to external biotic stimulus
response to external stimulus
AQP3
CGAS
RNASE6
CXCL5
CORO1A
TBXA2R
C3
HOPX
fold change
4
2
0
-2
size
13
14
16
17
C
NCOR2
NOTCH2
CIR1
NOTCH3
NOTCH1
LOC617905
LOC787465
MAML1
ATF6B
UBR4
HIST2H2BF
LOC505183
KAT2A
H2B
EGR3
TAF6
CCNH
LOC787269
HIST2H2BE
LOC104968456
DLG4
Notch signaling pathway
HIST3H2BB
CCNA1
ACTN1
TGFB1
BMP2
RELA
PXN
GTF2B
GTF2F1
SCRIB
LOC527388
BMP8B
GLI2
YWHAB
Viral carcinogenesis
LOC617875
GTF2A2
Basal transcription factors
TAF7
DVL3
HDAC5
H4
LIMD1
SMAD7
TLE3
ACTN4
GTF2H2
ERCC2
TEAD3
DVL2
EP300
CREBBP
HDAC4
HIST1H2BD
HIST1H2BJ
TAF9B
Hippo signaling pathway
JUN
CREB3L2
HDAC7
WWTR1
MYC
CSNK1E
PRKACA
NFKBIA
TAF4
SMAD2
LEF1
FBXW11
TCF7L1
LLGL1
Wnt signaling pathway
TCF7
WNT11
AXIN2
CSNK2A1
NFATC1
GPC4
PRICKLE2
PPARD
NFATC2
NOTUM
RUVBL1
LRP5
size
10
19
28
37
fold change
1
0
-1
-2
D
IFI16
GBP4
LOC781710
ZNF212
CYBA
LOC112441507
ZNF891
RHOA
TXNIP
NOD-like receptor signaling pathway
TAB2
ZNF317
ZNF45
LOC100336669
OAS1Z
OAS1Y
LOC100298356
OAS1X
CGAS
STAT1
IRF7
IRF9
Herpes simplex virus 1 infection
ZNF614
OAS2
C3
IKBKG
Measles
DDX58
TP53
Influenza A
EIF2AK2
DHX58
RIG-I-like receptor signaling pathway
IFIH1
RSAD2
MX2
TP73
ISG15
TNFSF10
IL1A
MX1
PRSS2
fold change
4
2
0
-2
-4
-6
-8
size
6
11
17
22

Supplementary File 3. List of matched genes (DEGs and top TF) between Brangus skin and leukocyte RNA-Seq studies. Table shows leukocyte dataset comparison (1 =match, blank = no match), whether genes is DEG and top-TF (by RIF), number of connections in each condition co-expression network (by PCIT).

Gene	Name	Number of connections		Category	DE in skin T12-vs-T0	DE in leukocytes		
		Low host resistance	High host resistance			T12-vs-T0	T3-vs-T0	LR-vs-HR (T0/T3/T12)
ACTL6A	actin like 6A	60	11	TF			1	
ADAMDEC1	ADAM-like, decysin 1	38	10	DEG			1	
ATRX	ATRX chromatin remodeler	68	6	TF		1	1	
BATF3	basic leucine zipper ATF-like transcription factor 3	27	13	TF		1	1	
BMI1	BMI1 proto-oncogene, polycomb ring finger	57	10	TF		1	1	
C3	complement C3	48	9	DEG		1	1	
CCNK	cyclin K	10	27	TF	1			
CCR3	C-C motif chemokine receptor 3	49	21	DEG		1	1	
CENPU	centromere protein U	32	17	TF	1			
COL4A2	collagen type IV alpha 2 chain	37	21	DEG	1			
CORO1A	coronin 1A	47	21	DEG			1	

Gene	Name	Number of connections		Category	DE in skin T12-vs-T0	DE in leukocytes		
		Low host resistance	High host resistance			T12-vs-T0	T3-vs-T0	LR-vs-HR (T0/T3/T12)
CPZ	carboxypeptidase Z	21	16	DEG			1	
CREB3L1	cAMP responsive element binding protein 3 like 1	71	32	DEG		1		
CREM	cAMP responsive element modulator	7	32	TF			1	
CYBA	cytochrome b-245 alpha chain	41	16	DEG			1	
CYP27A1	cytochrome P450, family 27, subfamily A, polypeptide 1	76	18	DEG		1	1	
DPF2	double PHD fingers 2	63	6	TF			1	
DVL3	dishevelled segment polarity protein 3	5	31	TF	1			
ELP1	elongator complex protein 1	41	30	TF		1	1	
FBXW7	F-box and WD repeat domain containing 7	9	30	TF		1	1	
FCMR	Fc fragment of IgM receptor	36	13	DEG				1
FCN1	ficolin 1	61	14	DEG		1	1	
FCRLA	Fc receptor like A	27	19	DEG			1	
FGR	FGR proto-oncogene, Src family tyrosine kinase	50	20	DEG			1	

Gene	Name	Number of connections		Category	DE in skin T12-vs-T0	DE in leukocytes		
		Low host resistance	High host resistance			T12-vs-T0	T3-vs-T0	LR-vs-HR (T0/T3/T12)
FMO5	flavin containing dimethylaniline monoxygenase 5	65	17	DEG			1	
GATA3	GATA binding protein 3	21	15	TF		1		
GIMAP5	GTPase, IMAP family member 5	33	25	DEG		1	1	
GPR82	G protein-coupled receptor 82	55	16	DEG		1		1
GPX7	glutathione peroxidase 7	50	32	DEG			1	
HJURP	Holliday junction recognition protein	47	5	DEG			1	
HLF	HLF transcription factor, PAR bZIP family member	44	26	TF		1		
HSPG2	heparan sulfate proteoglycan 2	77	25	DEG	1			
IL5RA	interleukin 5 receptor subunit alpha	56	17	DEG		1	1	
KDELR3	KDEL endoplasmic reticulum protein retention receptor 3	64	7	DEG		1	1	
KDM2B	lysine demethylase 2B	31	22	TF		1		
LOC104974401	uncharacterized LOC104974401	51	16	DEG				1

Gene	Name	Number of connections		Category	DE in skin T12-vs-T0	DE in leukocytes		
		Low host resistance	High host resistance			T12-vs-T0	T3-vs-T0	LR-vs-HR (T0/T3/T12)
LOC112447333	adhesion G protein-coupled receptor E2-like	25	11	DEG		1		
LOC508666	C-C motif chemokine 23	47	21	DEG			1	
LRPPRC	leucine rich pentatricopeptide repeat containing	44	9	TF	1		1	
LTB	lymphotoxin beta	57	11	DEG			1	
LTBP3	latent transforming growth factor beta binding protein 3	72	9	DEG	1		1	
LY6G6C	lymphocyte antigen 6 family member G6C	61	20	DEG			1	
MBD4	methyl-CpG binding domain 4, DNA glycosylase	16	20	TF		1	1	
MS4A1	membrane spanning 4-domains A1	64	22	DEG			1	
MYL9	myosin light chain 9	9	8	DEG		1		
NCOR1	nuclear receptor corepressor 1	48	24	TF			1	
NPAT	nuclear protein, coactivator of histone transcription	33	25	TF			1	
NRG1	neuregulin 1	69	4	TF			1	

Gene	Name	Number of connections		Category	DE in skin T12-vs-T0	DE in leukocytes		
		Low host resistance	High host resistance			T12-vs-T0	T3-vs-T0	LR-vs-HR (T0/T3/T12)
PDE4B	phosphodiesterase 4B	43	16	DEG		1		
PHF10	PHD finger protein 10	45	8	TF		1		
PRDM2	PR/SET domain 2	24	35	TF	1			
PRMT5	protein arginine methyltransferase 5	40	4	TF			1	
RBBP4	RB binding protein 4, chromatin remodeling factor	60	4	TF		1	1	
RCN1	reticulocalbin 1	48	22	DEG			1	
RETN	resistin	57	15	DEG		1	1	
RHOD	ras homolog family member D	44	17	DEG		1	1	
RIPOR2	RHO family interacting cell polarization regulator 2	63	9	DEG		1	1	
RNASE6	ribonuclease A family member k6	50	18	DEG			1	
SIRT2	sirtuin 2	37	7	TF		1		
SLC46A2	solute carrier family 46 member 2	54	21	DEG		1	1	
SLFN11	schlafen family member 11	38	25	DEG		1	1	
SNIP1	Smad nuclear interacting protein 1	55	9	TF		1	1	

Gene	Name	Number of connections		Category	DE in skin T12-vs-T0	DE in leukocytes		
		Low host resistance	High host resistance			T12-vs-T0	T3-vs-T0	LR-vs-HR (T0/T3/T12)
SOX4	SRY-box transcription factor 4	58	8	DEG			1	
SP3	Sp3 transcription factor	77	8	TF	1			
SP4	Sp4 transcription factor	70	7	TF		1		
SREBF2	sterol regulatory element binding transcription factor 2	49	30	TF		1	1	
TAF10	TATA-box binding protein associated factor 10	41	6	TF		1	1	
TBXA2R	thromboxane A2 receptor	74	16	DEG			1	
TEAD3	TEA domain transcription factor 3	82	17	TF	1			
TFAM	transcription factor A, mitochondrial	21	13	TF		1	1	
TFCP2L1	transcription factor CP2 like 1	31	17	TF	1			
THRB	thyroid hormone receptor beta	32	17	TF		1	1	
TMEM119	transmembrane protein 119	58	18	DEG			1	
TMEM263	transmembrane protein 263	66	33	DEG		1	1	

Gene	Name	Number of connections		Category	DE in skin T12-vs-T0	DE in leukocytes		
		Low host resistance	High host resistance			T12-vs-T0	T3-vs-T0	LR-vs-HR (T0/T3/T12)
TOB2	transducer of ERBB2, 2	11	31	TF	1	1	1	
TP53	tumor protein p53	8	21	TF			1	
TRAK2	trafficking kinesin protein 2	36	13	TF		1	1	
TUBA4A	tubulin alpha 4a	68	39	DEG		1	1	
TXNIP	thioredoxin interacting protein	39	17	TF			1	
VHL	von Hippel-Lindau tumor suppressor	49	7	TF		1	1	
WWP2	WW domain containing E3 ubiquitin protein ligase 2	50	29	TF			1	
ZBTB21	zinc finger and BTB domain containing 21	49	2	TF	1			
ZEB1	zinc finger E-box binding homeobox 1	13	33	TF		1	1	
ZGPAT	zinc finger CCCH-type and G-patch domain containing	11	10	TF			1	
ZNF397	zinc finger protein 397	26	18	TF	1			
ZNF644	zinc finger protein 644	60	3	TF			1	
